# Vasodilator reactive oxygen species ameliorate perturbed myocardial oxygen delivery in exercising swine with multiple comorbidities

**DOI:** 10.1007/s00395-024-01055-z

**Published:** 2024-05-25

**Authors:** R. W. A. van Drie, J. van de Wouw, L. M. Zandbergen, J. Dehairs, J. V. Swinnen, M. T. Mulder, M. C. Verhaar, A. MaassenVanDenBrink, D. J. Duncker, O. Sorop, D. Merkus

**Affiliations:** 1 Division of Experimental Cardiology, Department of Cardiology, Thoraxcenter, Erasmus University Medical Center, PO Box 2040, 3000 CA Rotterdam, The Netherlands; 2 Laboratory of Vascular Medicine, Department of Internal Medicine, Erasmus University Medical Center, Rotterdam, The Netherlands; 3 Laboratory of Lipid Metabolism and Cancer, Department of Oncology, KU Leuven—University of Leuven, Leuven, Belgium; 4 Department of Nephrology and Hypertension, University Medical Center Utrecht, Utrecht, The Netherlands; 5 Walter Brendel Center of Experimental Medicine (WBex), University Clinic Munich, 81377 LMU Munich, Germany; 6 Center for Cardiovascular Research (DZHK), Munich Heart Alliance (MHA), Partner Site Munich, 81377 Munich, Germany; 7 Interfaculty Center for Endocrine and Cardiovascular Disease Network Modelling and Clinical Transfer (ICONLMU), University Clinic Munich, LMU, Munich, Germany

**Keywords:** Reactive oxygen species, Swine, Myocardial perfusion, Coronary microcirculation, Metabolic syndrome

## Abstract

Multiple common cardiovascular comorbidities produce coronary microvascular dysfunction. We previously observed in swine that a combination of diabetes mellitus (DM), high fat diet (HFD) and chronic kidney disease (CKD) induced systemic inflammation, increased oxidative stress and produced coronary endothelial dysfunction, altering control of coronary microvascular tone via loss of NO bioavailability, which was associated with an increase in circulating endothelin (ET). In the present study, we tested the hypotheses that (1) ROS scavenging and (2) ET_A+B_-receptor blockade improve myocardial oxygen delivery in the same female swine model. Healthy female swine on normal pig chow served as controls (Normal). Five months after induction of DM (streptozotocin, 3 × 50 mg kg^−1^ i.v.), hypercholesterolemia (HFD) and CKD (renal embolization), swine were chronically instrumented and studied at rest and during exercise. Sustained hyperglycemia, hypercholesterolemia and renal dysfunction were accompanied by systemic inflammation and oxidative stress. In vivo ROS scavenging (TEMPOL + MPG) reduced myocardial oxygen delivery in DM + HFD + CKD swine, suggestive of a vasodilator influence of endogenous ROS, while it had no effect in Normal swine. In vitro wire myography revealed a vasodilator role for hydrogen peroxide (H_2_O_2_) in isolated small coronary artery segments from DM + HFD + CKD, but not Normal swine. Increased catalase activity and ceramide production in left ventricular myocardial tissue of DM + HFD + CKD swine further suggest that increased H_2_O_2_ acts as vasodilator ROS in the coronary microvasculature. Despite elevated ET-1 plasma levels in DM + HFD + CKD swine, ET_A+B_ blockade did not affect myocardial oxygen delivery in Normal or DM + HFD + CKD swine. In conclusion, loss of NO bioavailability due to 5 months exposure to multiple comorbidities is partially compensated by increased H_2_O_2_-mediated coronary vasodilation.

**Supplementary Information:**

The online version contains supplementary material available at 10.1007/s00395-024-01055-z.

## Introduction

Myocardial ischemia in the absence of significant coronary artery stenosis is proposed to be caused by coronary microvascular dysfunction (CMD) and is associated with adverse cardiovascular outcome [1, 9, 39, 40, 42, 49]. The exact mechanisms underlying the development of CMD are currently unknown. However, CMD is predominantly observed in women [1, 80, 86] and is more prevalent in patients with cardiovascular risk factors including diabetes mellitus (DM), hypercholesterolemia and chronic kidney disease (CKD) [65, 87]. Multiple studies in humans and animal models have shown that these cardiovascular risk factors can induce a systemic pro-inflammatory state associated with increased production of reactive oxygen species (ROS) [29, 66, 78, 83]. In the healthy vasculature, ROS have important cell signaling functions, and their excessive production is counterbalanced by various antioxidant systems [81]. When the balance between antioxidants and ROS production is disturbed, ROS production overwhelms antioxidant capacity, resulting in oxidative stress. Oxidative stress has been shown to contribute to the development of ischemia with no occlusive coronary artery disease (INOCA) [64, 70]. Potential sources of ROS production in the myocardium are the nicotinamide adenine dinucleotide phosphate (NADPH) oxidases, mitochondria, xanthine oxidase and uncoupled nitric oxide synthase (NOS) [23, 81]. Furthermore, increased intracellular ROS have also been shown to affect vasomotor tone regulation, by inducing direct vasoconstriction (superoxide) or vasodilation (H_2_O_2_), as well as by scavenging nitric oxide (NO) [50, 51, 59, 75, 76, 81, 82]. Additionally, loss of NO, inflammation and increased ROS production have also been linked to NADPH oxidase activation by increases in vasoconstrictor endothelin-1 (ET-1) [47, 53, 69].

Recently, we demonstrated that female swine exposed for 5 months to DM, high fat diet (HFD) and chronic kidney disease (CKD) developed CMD in the absence of appreciable atherosclerosis [89]. CMD was evidenced by reduced coronary flow reserve and perturbations in myocardial oxygen delivery, particularly during exercise [89]. The perturbations in oxygen balance were not related to structural alterations in the microvasculature but were accompanied by reduced coronary NO bioavailability, increased oxidative stress and increased circulating levels of ET-1 [88, 90]. In the present study, we therefore tested the hypotheses that (1) ROS scavenging and (2) ET-receptor blockade restore microvascular function and myocardial oxygen delivery. To test these hypotheses, first, we assessed the effects of ROS scavenging on the myocardial oxygen delivery in vivo and studied contributions of specific ROS on small artery vasomotor function in vitro. Second, we assessed whether ET-1 exerted an enhanced vasoconstrictor influence on the coronary microvasculature in DM + HFD + CKD in vivo. Our results point toward a role for a vasodilator ROS that compensates for the reduced bioavailability of NO. Since accumulation of ceramides was associated with mitochondrial H_2_O_2_ production in the coronary microvasculature of patients with coronary artery disease [30], we also investigated if circulating ceramides as well as factors involved in ceramide metabolism were altered in swine with DM + HFD + CKD.

## Materials and methods

### In vivo experimental protocol

#### Animals

All animal experiments were approved by the Netherlands National Committee for the Protection of Animals used for Scientific Purposes (license number AVD1010020185224) as well as by the Animal welfare body at the Erasmus University Medical Center (Rotterdam, The Netherlands) and performed in accordance with the guidelines from Directive 2010/63/EU of the European Parliament on the protection of animals used for scientific purposes. In light of the higher prevalence of CMD in women [1, 80, 86], given differences in mechanisms controlling coronary microvascular tone [14, 15] and because our previous studies, which form the basis of the present study, were performed in female swine [88, 89], we elected to perform the present study in female swine. In total, 38 female Yorkshire × Landrace swine entered the study: 21 female swine (24.4 ± 0.9 kg, ~ 3 months of age) were included in the DM + HFD + CKD group, while 18 healthy female swine of similar age and weight were used as controls (Normal). Several animals have also been included in our previous studies [88, 89]; however, the data presented here are new and original and have not been published previously.

#### Risk factors

Cardiovascular risk factors were induced as previously described [78, 89]. Briefly, diabetes mellitus (DM) was induced by destruction of  ~ 80% of insulin- producing cells in the pancreas using bolus injections of streptozotocin (50 μg kg^−1^ day^−1^ i.v., AdipoGen Life Sciences, Inc., San Diego, CA, USA) for three consecutive days. This results in a type 2-like DM phenotype, with swine being hyperglycemic, but insulin-independent [89].

Two weeks after DM induction, chronic kidney disease (CKD) was produced by renal microembolization with microspheres (38–42 μm, Cospheric, Santa Barbara, CA, USA). The animals were sedated with an intramuscular injection of a cocktail of Zoletil (tiletamine/zolazepam; 5 mg kg^−1^), Sedazine (xylazine; 2.25 mg kg^−1^) and atropine (1 mg), intubated and artificially ventilated with a mixture of O_2_ and N_2_ (1:2 vol/vol), to which 1–2% (vol/vol) sevoflurane was added for anesthesia. Fluoroscopy-guided catheterization of the renal arteries was performed using a 7F Swan Ganz catheter. Microspheres (~ 75 mg, 2 × 10^6^ microspheres) were suspended in heparinized autologous blood and injected in the right renal artery and in the inferior branch of the left renal artery through the distal port of the catheter, while backflow into the aorta was prevented by inflation of the balloon.

Hypercholesterolemia was induced using a high fat, high sugar diet (high fat diet, HFD) (10% sucrose, 15% fructose, 25% saturated fats and 1% cholesterol, Research Diet Services BV, Wijk bij Duurstede, The Netherlands) supplemented with 20 g day^−1^ sodium chloride. Animals in the Normal group were fed regular swine chow. All animals were housed in pairs, but were fed separately and had ad libitum access to fresh drinking water.

#### Surgical instrumentation

Five months after introduction of the risk factors, 21 DM + HFD + CKD swine and 18 Normal swine (101 ± 3 kg vs. 97 ± 5 kg, *p* = NS) were chronically instrumented as previously described [21]. In short, the animals were sedated with an intramuscular injection of Zoletil (tiletamine/zolazepam; 5 mg kg^−1^), Sedazine (xylazine; 2.25 mg kg^−1^) and atropine (2 mg), intubated and artificially ventilated with a mixture of O_2_ and N_2_ (1:2 vol/vol), to which 1–2% (vol/vol) sevoflurane was added for anesthesia. Additionally sufentanil i.v. (5 μg kg^−1^ h^−1^) was used for peri-thoracotomy analgesia. Thoracotomy was performed in the fourth left intercostal space and polyvinylchloride catheters were placed in the aorta, left ventricle, pulmonary artery and left atrium for measurement of pressure and blood sampling. Additionally, two custom-made small angiocatheters were placed in the anterior interventricular coronary vein for blood sampling and flow probes were placed around the aorta and left anterior descending coronary artery (Transonic Systems Inc., Ithaca, NY, USA). All catheters and electrical wires were tunneled subcutaneously to exit through the back of the animal. Then the chest was closed and the implanted catheters were protected with a stretchable vest (Tensogrip, BSN medical/Essity, Stockholm, Sweden). Animals were allowed to recover, receiving analgesia (0.3 mg buprenorphine i.m.) and a slow-release fentanyl patch (50 μg h^−1^) for 6 days, and antibiotic prophylaxis (25 mg kg^−1^ amoxicillin i.v.) for 7 days. All fluid-filled catheters were flushed daily and filled with fresh heparinized saline (1000–5000 IU ml^−1^) to maintain catheter patency. Two swine in the Normal group and two swine in the DM + HFD + CKD group died prematurely due to complications after surgery.

#### Awake hemodynamic measurements

Experiments were performed on a motor-driven treadmill, modified for swine. The experimental protocol included continuous hemodynamic measurements on a Codas workstation (ATCODAS, Dataq Instruments, Akron, OH, USA), and blood sampling for pO_2_, pCO_2_, pH, bicarbonate, hemoglobin concentrations and its O_2_ saturation (sO_2_), lactate and pH analysis (ABL-800, Radiometer, Copenhagen, Denmark) from the aorta and coronary vein in resting state and in the last 30 s of each 3-min exercise stage (2, 3 and 4 km h^−1^ at 0% incline). Unfortunately, coronary vein catheters lost patency in one swine in the Normal group and five swine in the DM + HFD + CKD group before all exercise experiments could be completed, and one swine from the DM + HFD + CKD group had a malfunctioning coronary flow probe. The exercise protocols were conducted under control conditions as well as in the presence of ROS-scavenging or dual endothelin-1 receptors A and B (ET_A_/ET_B_) blockade as described below. Multiple control exercise trials were conducted in each pig, and each exercise trial in the presence of blockers was matched to the nearest control exercise experiment (performed within 48 h).

The exercise protocol in the presence of ROS scavengers (10 Normal and 11 DM + HFD + CKD swine) was conducted as previously described [90]. Briefly, ROS scavenging was achieved by continuous infusion of 1 mg kg^−1^ min^−1^ of free radical scavenger N-(2-mercaptopropionyl)glycine (MPG, Sigma-Aldrich, Zwijndrecht, The Netherlands) and a bolus infusion of 30 mg kg^−1^ i.v. of superoxide dismutase mimetic 4-hydroxy-2,2,6,6-tetramethylpiperidine-N-oxyl (TEMPOL, Sigma-Aldrich) over 10 min. Upon completion of the bolus TEMPOL, hemodynamic measurement and blood samples were obtained at rest and the exercise protocol was started.

On another day, the exercise protocol was repeated during dual ET_A_ and ET_B_ receptor blockade in 9 Normal and 11 DM + HFD + CKD swine, achieved by intravenous infusion of the mixed ET_A_/ET_B_ receptor blocker tezosentan (a gift from Actelion Pharmaceuticals Ltd, Allschwil, Switzerland) bolus of 3 mg kg^−1^, followed by a continuous infusion of 100 µg kg^−1^ min^−1^ [90].

Coronary flow reserve (CFR) was measured, in awake resting swine (4 Normal and 8 DM + HFD + CKD swine). Maximal coronary blood flow was achieved by intravenous infusion of adenosine (0.5 mg kg^−1^ min^−1^) while intravenous infusion of the systemic vasoconstrictor phenylephrine (5–7.5 µg kg^−1^ min^−1^) was titrated to stabilize mean arterial blood pressure.

#### Termination

For termination, swine were sedated as described above and anesthetized with i.v. pentobarbital (9 mg kg^−1^ min^−1^) and were artificially ventilated with a mixture of O_2_ and N_2_ (1:2 vol/vol). Thereafter, ventricular fibrillation was induced and ventilation stopped. The heart was excised, weighed and prepared for later biochemical, molecular and histological analyses. Coronary small arteries were isolated for in vitro coronary small artery functional experiments. One swine in the DM + HFD + CKD group had a myocardial infarction in the last days prior to termination as a consequence of a twisted flow probe around the left anterior descending coronary artery. Therefore, data of this animal gathered after the infarction were excluded from all analyses.

### In vitro coronary small artery function

Coronary small arteries (∼300 µm diameter) were isolated from the epicardial surface of the left ventricular apex and studied in vitro using a Mulvany wire myography (DMT, Aarhus, Denmark) as earlier described [78] (6 Normal and 13 DM + HFD + CKD swine). Vasodilation to the endothelium-dependent vasodilator bradykinin (BK, 10^−10^–10^−6^ mol L^−1^, Sigma–Aldrich) was measured following precontraction with 10^−6^ mol L^−1^ of the thromboxane-A_2_ analog U46619 (Sigma-Aldrich). On adjacent vascular segments, the concentration–response curves for bradykinin were also performed in the presence of the ROS scavengers MPG (10^−5^ mol L^−1^, Sigma-Aldrich) and TEMPOL (10^−3^ mol L^−1^, Sigma-Aldrich). Additionally, on small coronary arteries isolated from 5 Normal and 9 DM + HFD + CKD animals concentration–response curves for bradykinin were also attained in the presence of MPG + TEMPOL as well as catalase (10^3^ U mL^−1^, Sigma-Aldrich) to assess the contribution of H_2_O_2_.

### Biochemical and molecular assays

At the time of instrumentation (5 months follow-up), fasting arterial blood samples were obtained and analyzed by clinical routine standard methods for plasma glucose, triglycerides, total cholesterol, low-density lipoprotein (LDL), high-density lipoprotein (HDL), albumin and creatinine. Thiobarbituric acid reactive substances (TBARS) assay (Cayman Chemical, Ann Arbor, MI, USA) was used according to the manufacturer’s instructions for analyzing lipid peroxidation. Aldosterone was assayed using a RIA-kit (Beckman Coulter, Brea, CA, USA). The concentration of active plasma renin was measured by enzyme-kinetic assay as described before [17, 19]. The minimum detectable levels for aldosterone and renin assays were 12 pmol ml^−1^ and 0.17 ng Ang I ml^−1^ per hour, respectively. If measurements fell at or below these thresholds, the measurement was set to this threshold.

The total concentration of sphingomyelins and ceramides was determined by Lipometrix, in the lipidomics core facility at KU Leuven, using HILIC LC–MS/MS as previously described [16]. In short, lipids were extracted according to a modified Bligh and Dyer protocol with the addition of an internal standard mix (3 μl of SPLASH® LIPIDOMIX® Mass Spec Standard (#330707, Avanti Polar Lipids)). The extract was analyzed with a targeted lipidomics method, using HILIC separation (with a XBridge amide column (150 mm × 4.6 mm, 3.5 μm; Waters)) and an MRM assay on a 6500 + QTRAP mass spectrometer (AB SCIEX).

Snap- frozen bulk left ventricular subendocardial tissue samples were used for measuring myocardial catalase activity (Cayman Chemical), total glutathione (BioVision, Milpitas, CA, USA), total 8-isoprostane (Cayman Chemical) as well as total myocardial antioxidant capacity (Total Antioxidant Capacity Assay kit, Abcam plc., Cambridge, UK). Total antioxidant capacity was reported previously in [88].

Total RNA was isolated from  ~ 30 mg snap-frozen left ventricular subendocardial tissue using the ISOLATE II RNA Mini Kit (Bioline, London, UK) according to manufacturer’s instructions with addition of proteinase K (Invitrogen, Carlsbad, CA, USA) treatment at 55 °C for 10 min. After RNA purity and concentration measurement, cDNA was synthesized (SensiFAST cDNA synthesis kit, Bioline) using 500 ng RNA. Quantitative polymerase chain reaction was performed using the CFX96 Real-Time PCR detection system (Bio-Rad, Hercules, CA, USA) and the SensiMix SYBR-green supermix (Bioline). Gene specific primers were designed using the online tools Primer3 and NCBI primer BLAST, thereafter synthesized (IDT, Coralville, IA, USA) and tested for gene specificity by gel electrophoresis and qPCR dilution series. Primers used for qPCR are listed in Supplementary Table 1. Quality control and analyses of gene expression data were executed using the Bio-Rad software (Bio-Rad CFX manager 3.1). All results were normalized to hypoxanthine phosphoribosyl transferase 1 (HPRT1) and ribosomal protein L13 (RPL13a) as housekeeping genes.

### Data analysis and statistics

Digital recording and off-line analysis of hemodynamic data have been described in detail previously [89]. Myocardial oxygen delivery (MDO_2_) was computed as the product of LAD coronary blood flow and arterial blood O_2_ content. Myocardial oxygen consumption (MVO_2_) in the LAD perfused area was computed as the product of LAD coronary blood flow and the difference in O_2_ content between arterial and coronary venous blood. Myocardial O_2_ extraction (MEO_2_) was computed as MVO_2_/MDO_2_ • 100%. The rate–pressure product (RPP) was computed as the product of heart rate and systolic aortic blood pressure. Coronary blood flow, MDO_2_ and MVO_2_ were normalized per gram of myocardium perfused by the LAD, which was estimated to be 40% of the left ventricle. Coronary vascular conductance (CVC) was calculated as the ratio of normalized coronary blood flow and mean arterial pressure. CFR was calculated as the ratio of maximal coronary blood flow (adenosine) and resting coronary blood flow.

Statistical analysis was performed using SPSS Statistics 21.0 (IBM Corp, Armonk, NY, USA). Single time point variables were tested for normality using the Shapiro–Wilk and Kolmogorov–Smirnov test. Data showing a normal distribution are presented as mean ± SEM, whereas data without normal distribution are shown as median [interquartile range (IQR)]. Comparison between the two groups was performed by unpaired Student’s *t* test for parametric data or Mann–Whitney *U* test for non-parametric data. In vitro coronary microvascular responses to pharmacological agents were analyzed using two-way ANOVA for repeated measures. In vivo hemodynamics and myocardial oxygen delivery responses to exercise and intervention were tested using three-way followed by two-way ANOVA and ANCOVA for repeated measures, followed by post hoc testing when appropriate, using least significant difference correction. Statistical significance was accepted when *P* < 0.05 (two-tailed).

## Results

### In vivo coronary vascular function

Five months exposure to the combination of diabetes mellitus (DM), chronic kidney disease (CKD) and high fat diet (HFD) led to pronounced hyperglycemia, hypercholesterolemia and reduced renal function in female swine, as evidenced by increased plasma creatinine concentrations in the DM + HFD + CKD group (Table 1). Plasma aldosterone and renin were not different, suggesting the absence of alterations in the renin–angiotensin–aldosterone system 5 months after the kidney embolization (Table 1). Resting coronary blood flow (CBF) was higher in the DM + HFD + CKD animals, while maximal CBF (intravenous infusion of adenosine + phenylephrine to negate changes in mean arterial pressure) was unchanged, resulting in a significantly lower coronary flow reserve in the DM + HFD + CKD group (Table 2).

**Table 1:** Plasma metabolic parameters of Normal and DM + HFD + CKD swine

	Normal	*n*	DM + HFD + CKD	*n*
*Blood profile*				
Glucose (mmol L^−1^)	8.5 ± 0.7	13	18.1 ± 1.0*	18
Triglycerides (mmol L^−1^)	0.20 ± 0.02	13	0.72 ± 0.30	18
Cholesterol (mmol L^−1^)	1.7 ± 0.1	13	12.2 ± 2.0*	18
LDL (mmol L^−1^)	1.03 ± 0.04	13	10.46 ± 1.90*	18
HDL (mmol L^−1^)	0.88 ± 0.04	13	2.52 ± 0.31*	18
LDL/HDL ratio	1.21 ± 0.06	13	5.89 ± 1.95*	18
Creatinine (μmol L^−1^)	119 ± 3	13	165 ± 7*	18
Endothelin-1 (pg ml^−1^)	29 ± 2	10	36 ± 2*	8
Aldosterone (pg mL^−1^)	12 (12–18)	9	12 (12–12)	15
Renin (ng Ang I mL^−1^ h^−1^)	0.17 (0.17–2.32)	9	0.58 (0.17–1.08)	15

*LDL* low density lipoprotein, *HDL* high density lipoprotein. Values are mean ± SEM or median (IQR). *P ≤ 0.05 Normal vs. DM + HFD + CKD by unpaired Student’s *t*-test

**Table 2:** Hemodynamics in Normal and DM + HFD + CKD swine in the absence and presence of adenosine

		*n*	Basal	Adenosine	Adenosine/Basal
Heart rate (beats min^ − 1^)	Normal	4	113 ± 12	114 ± 4	1.03 ± 0.10
	DM + HFD + CKD	8	109 ± 6	103 ± 4	0.96 ± 0.05
MAP (mmHg)	Normal	4	96 ± 5	92 ± 5*	0.96 ± 0.01
	DM + HFD + CKD	8	94 ± 3	90 ± 3*	0.95 ± 0.01
CI (mL min^−1^ kg^−1^)	Normal	4	61 ± 10	61 ± 13	1.00 ± 0.10
	DM + HFD + CKD	7	104 ± 9†	95 ± 8†	0.93 ± 0.06
RPP (10^−2^ [mmHg beats min^−1^])	Normal	4	113 ± 8	110 ± 1	0.99 ± 0.07
	DM + HFD + CKD	7	102 ± 5	92 ± 3†	0.91 ± 0.04
CBF (ml min^−1^ g^−1^)	Normal	4	0.72 ± 0.09	2.61 ± 0.31*	3.64 ± 0.24
	DM + HFD + CKD	8	1.04 ± 0.07†	2.74 ± 0.20*	2.69 ± 0.21†
CVC (μL mmHg^−1^ min^−1^ g^−1^)	Normal	4	7.5 ± 1.0	28.9 ± 4.6*	3.80 ± 0.25
	DM + HFD + CKD	8	11.1 ± 0.9†	30.7 ± 2.1*	2.84 ± 0.23†

*MAP* mean arterial pressure, *CI* cardiac index, *RPP* rate pressure product, *CBF* coronary blood flow per gram of myocardium, *CVC* coronary vascular conductance. Values are mean ± SEM. *P ≤ 0.05 versus basal by paired Student’s *t* test; ^†^*P* ≤ 0.05 versus corresponding Normal by unpaired Student’s *t* test

**Fig. 1:**
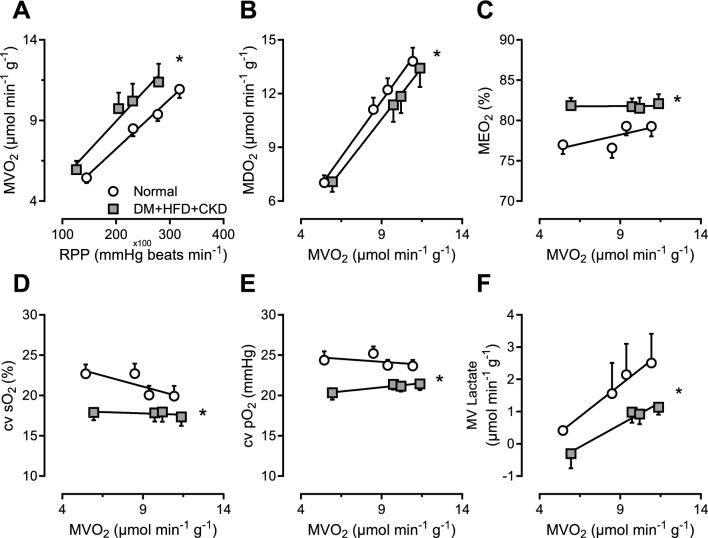
In vivo myocardial oxygen balance at rest and during exercise in Normal and DM + HFD + CKD swine. *RPP* rate–pressure product, *MDO*_*2*_ myocardial oxygen delivery, *MVO*_*2*_ myocardial oxygen consumption, *MEO*_*2*_ myocardial oxygen extraction, *cv sO*_*2*_ coronary venous oxygen saturation, *cv pO*_*2*_ coronary venous partial oxygen pressure, *MV* Lactate, lactate consumption per gram of myocardium. Values are mean ± SEM. *P ≤ 0.05 by two-way ANCOVA for repeated measures. Normal *n* = 11, DM + HFD + CKD *n* = 13

**Fig. 2:**
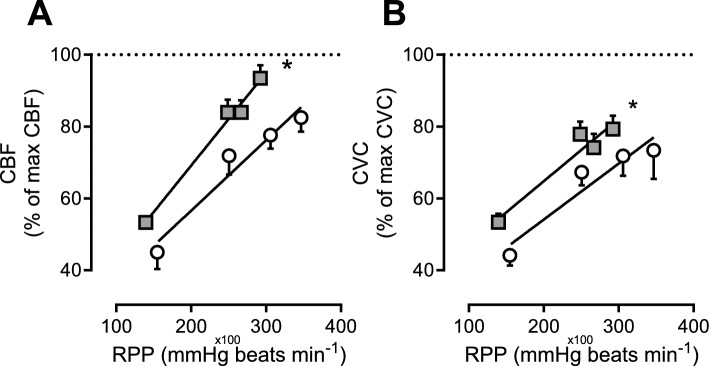
In vivo coronary blood flow and conductance at rest and during exercise in Normal and DM + HFD + CKD swine relative to individually matched maximal coronary blood flow and coronary vascular conductance during maximal vasodilation with adenosine. CBF, coronary blood flow; CVC, coronary vascular conductance; RPP, rate–pressure product. Values are mean ± SEM. *P ≤ 0.05 by two-way ANCOVA for repeated measures. Normal *n* = 3, DM + HFD + CKD *n* = 5

In vivo hemodynamic measurements during graded treadmill exercise revealed higher myocardial oxygen consumption (MVO_2_) at similar levels of myocardial work (RPP) in the DM + HFD + CKD group (Fig. 1A, Table 3), suggestive of reduced myocardial oxygen utilization efficiency. Since myocardial oxygen delivery was lower at each level of MVO_2_ (Fig. 1B), the higher MVO_2_ was accomplished by a higher myocardial oxygen extraction (MEO_2_) (Fig. 1C), which reduced the coronary venous oxygen saturation (cv sO_2_) and coronary venous oxygen tension (cv pO_2_) (Fig. 1D, E). Furthermore, maximal CBF in DM + HFD + CKD swine during exercise was similar to maximal CBF in the presence of adenosine (Fig. 2A), which, together with the higher MEO_2_ and lower lactate consumption rate (Fig. 1F) during exercise, is indicative of the exhaustion of coronary flow reserve during exercise. In Normal swine, maximal CBF during exercise remained lower than maximal CBF in the presence of adenosine (Fig. 2A), indicating that there was vasodilator reserve left. In both Normal and DM + HFD + CKD swine, CVC showed only minor changes between exercise at 2 and 4 km h^−1^ (Fig. 2B), indicating that the increase in CBF at higher levels of exercise was driven in part by a modest increase in mean arterial pressure (Table 2).

**Table 3:** Hemodynamics at rest and during exercise under control conditions and after ROS scavenging in Normal and DM + HFD + CKD swine

			Standing		Exercise			ANOVA *P* value		
			*n*		2 km h^−1^	3 km h^−1^	4 km h^−1^	MPG + TEMPOL	Group	MPG + TEMPOL *Group
Heart rate (beats min^−1^)	Normal	Control	10	117 ± 5	117 ± 13*	213 ± 13*	235 ± 10*			
		MPG + TEMPOL	10	142 ± 8‡	198 ± 11*	224 ± 10*	241 ± 12*	0.249		
	DM + HFD + CKD	Control	11	125 ± 5	176 ± 11*	193 ± 9*	206 ± 9*		0.072	
		MPG + TEMPOL	11	149 ± 8‡	187 ± 9*	204 ± 9*	218 ± 11*	0.266	0.103	0.804
MAP (mmHg)	Normal	Control	10	87 ± 3	91 ± 3	96 ± 3	100 ± 3*			
		MPG + TEMPOL	10	80 ± 3	85 ± 2	90 ± 2	92 ± 4	0.003		
	DM + HFD + CKD	Control	11	87 ± 3	92 ± 3	93 ± 3	96 ± 4		0.400	
		MPG + TEMPOL	11	73 ± 5†‡	79 ± 4†‡	85 ± 3	87 ± 2*	0.001	0.027	0.301
CI (mL min^−1^ kg^−1^)	Normal	Control	10	136 ± 7	198 ± 8	208 ± 12*	228 ± 12*			
		MPG + TEMPOL	10	136 ± 10‡	200 ± 16	223 ± 16*	244 ± 13*	0.590		
	DM + HFD + CKD	Control	8	130 ± 11	184 ± 17	191 ± 15*	218 ± 20*		0.147	
		MPG + TEMPOL	8	143 ± 11	185 ± 15	198 ± 14*	223 ± 17*	0.597	0.086	0.768
CBF (ml min^−1^ g^−1^)	Normal	Control	10	1.09 ± 0.07	1.63 ± 0.09*	1.86 ± 0.11*	2.09 ± 0.12*			
		MPG + TEMPOL	10	1.28 ± 0.07	1.65 ± 0.08*	1.91 ± 0.12*	1.94 ± 0.14*	0.622		
	DM + HFD + CKD	Control	8	1.31 ± 0.09	1.92 ± 0.18*	1.95 ± 0.17*	2.09 ± 0.09*		0.288	
		MPG + TEMPOL	8	1.31 ± 0.14	1.71 ± 0.09	1.89 ± 0.13*	2.00 ± 0.10*	0.117	0.630	0.579
Haemoglobin (g dl^−1^)	Normal	Control	10	9.4 ± 0.4	10.4 ± 0.4	11.0 ± 0.6	11.4 ± 0.6			
		MPG + TEMPOL	10	9.8 ± 0.7	11.6 ± 1.0	10.7 ± 0.8	11.7 ± 0.9	0.443		
	DM + HFD + CKD	Control	11	9.3 ± 0.4	10.2 ± 0.4	10.3 ± 0.5	11.4 ± 0.5		0.751	
		MPG + TEMPOL	11	9.2 ± 0.6	10.4 ± 0.6	11.2 ± 0.5	11.4 ± 0.4	0.193	0.964	0.820
Arterial sO_2_ (%)	Normal	Control	10	98 ± 1	97 ± 1	97 ± 1	97 ± 1			
		MPG + TEMPOL	10	100 ± 1	96 ± 1*	98 ± 1	97 ± 1*	0.743		
	DM + HFD + CKD	Control	11	98 ± 1	97 ± 1	96 ± 1	97 ± 1		0.139	
		MPG + TEMPOL	11	97 ± 1†	97 ± 1	97 ± 1	97 ± 1	0.151	0.417	0.115
MVO_2_ (μmol min^−1^ g^−1^)	Normal	Control	8	5.1 ± 0.5	7.9 ± 0.5*	10.5 ± 1.0*	11.9 ± 1.0*			
		MPG + TEMPOL	8	6.2 ± 0.6	9.1 ± 1.0	10.6 ± 1.1*	11.2 ± 1.3*	0.032		
	DM + HFD + CKD	Control	5	6.0 ± 0.7	8.6 ± 1.7	9.8 ± 1.5	12.1 ± 1.6*		0.772	
		MPG + TEMPOL	5	6.2 ± 1.2	9.0 ± 0.9	10.3 ± 0.9	11.4 ± 0.8*	0.160	0.882	0.755
RPP (10^−2^ [mmHg beats min^−1^])	Normal	Control	10	131 ± 7	217 ± 17*	282 ± 20*	328 ± 17*			
		MPG + TEMPOL	10	151 ± 10	239 ± 18*	286 ± 17*	314 ± 25*	0.362		
	DM + HFD + CKD	Control	11	139 ± 7	220 ± 18*	248 ± 15*	270 ± 14*†		0.087	
		MPG + TEMPOL	11	148 ± 13	207 ± 17*	246 ± 15*	279 ± 19*	0.316	0.039	0.828

*MAP* mean arterial pressure, *CI* cardiac output per kg of bodyweight, *CBF* coronary blood flow per gram of myocardium, *sO*_*2*_ oxygen saturation, *MVO*_*2*_ myocardial oxygen consumption, *RPP* rate pressure product. Values are mean ± SEM. *P ≤ 0.05 versus corresponding standing; †*P* ≤ 0.05 versus corresponding Normal; ‡*P* ≤ 0.05 versus corresponding Control by three-way ANOVA for repeated measures and post-hoc analysis with least significant difference correction

### Myocardial reactive oxygen species

**Fig. 3:**
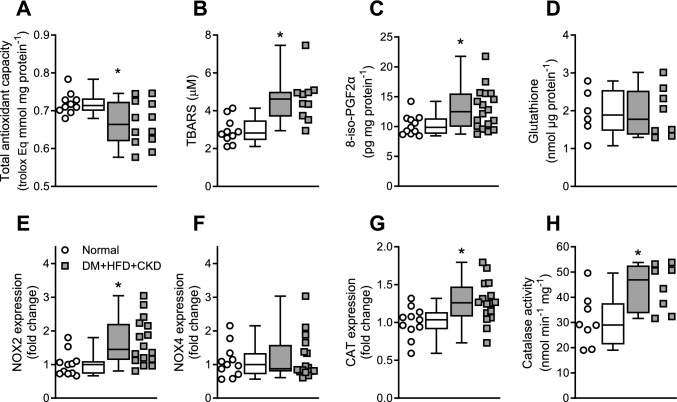
Measures for oxidative stress in left ventricular (LV) tissue and plasma of Normal and DM + HFD + CKD swine. **A** Total antioxidant capacity in LV tissue, **B** thiobarbituric acid reactive substances (TBARS) in plasma, **C** 8-isoprostane (8-iso-PGF2α) in plasma, **D** total glutathione in LV tissue, **E** LV mRNA expression as fold change relative to Normal of nicotinamide adenine dinucleotide phosphate (NADPH) oxidase 2 (NOX2), **F** NOX4 and **G** catalase (CAT), **H** catalase activity, catalase activity in LV tissue. *P ≤ 0.05 Normal vs. DM + HFD + CKD

Myocardial reactive oxygen species were further assessed to establish whether decreased oxygen utilization efficiency was due to an increase in myocardial ROS. Total myocardial antioxidant capacity was lower in DM + HFD + CKD swine (Fig. 3A, and previously published in [88]). Plasma oxidized lipids (TBARS) and bulk myocardium 8-isoprostane (8-iso-PGF2α) concentrations were significantly higher in the DM + HFD + CKD swine, all indicative of increased presence of ROS (Fig. 3B, C). In vitro analyses were performed to further elucidate the production and breakdown of myocardial ROS in the DM + HFD + CKD swine; data are presented in Fig. 3 and Supplementary Table 2. The mRNA expression of nicotinamide adenine dinucleotide phosphate (NADPH) oxidase 2 (NOX2) in bulk left ventricular tissue was higher in the DM + HFD + CKD swine (Fig. 3E) indicating that NOX2 is a potential source of increased ROS production. The gene expressions of NADPH oxidase 4 (NOX4, Fig. 3F) and xanthine dehydrogenase (XDH, Supplementary Table 2) were not altered compared to Normal swine. Although the expressions of superoxide dismutases (SOD1, SOD2, SOD3) were not significantly altered, catalase (CAT) gene expression was increased in DM + HFD + CKD, which was accompanied by a higher catalase activity (Fig. 3G, H). Expression of glutathione peroxidase 1 (GPX, Supplementary Table 2) and the total concentration of glutathione (GSH + GSSG) were not significantly different between groups (Fig. 3D).

### Scavenging of reactive oxygen species during exercise

**Fig. 4:**
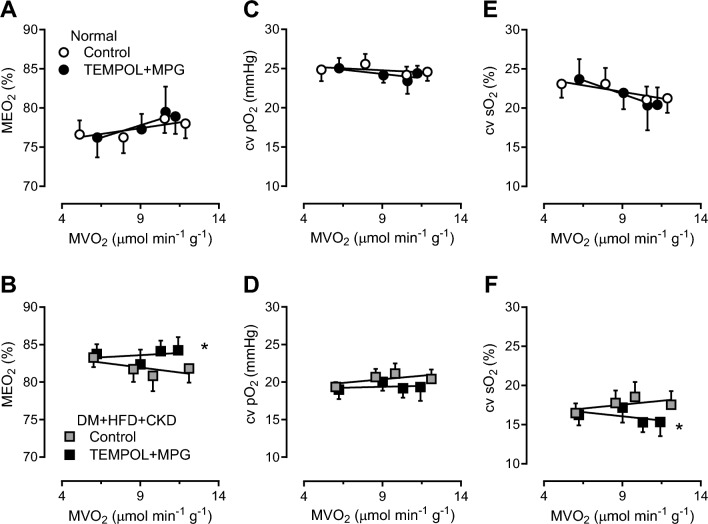
In vivo effects of ROS scavenging (MPG + TEMPOL) on myocardial oxygen balance in rest and during exercise in Normal and DM + HFD + CKD swine. *MDO*_*2*_ myocardial oxygen delivery, *MVO*_*2*_ myocardial oxygen consumption, *MEO*_*2*_ myocardial oxygen extraction, *cv sO*_*2*_ coronary venous oxygen saturation, *cv pO*_*2*_ coronary venous partial oxygen pressure. Values are mean ± SEM. *P ≤ 0.05 by two-way ANCOVA for repeated measures. Normal *n* = 5, DM + HFD + CKD *n* = 8

To test our hypothesis that ROS scavenging would result in coronary vasodilation, thereby restoring myocardial oxygen balance, graded treadmill exercise experiments were performed in the absence and presence of the superoxide scavenger MPG and SOD-mimetic TEMPOL in both groups. Cardiac output was unaltered in response to ROS scavenging, while ROS scavenging reduced the mean arterial pressure in both groups, but more pronounced in DM + HFD + CKD (Table 3), consistent with our previous findings. Under control conditions, DM + HFD + CKD animals showed a higher MEO_2_ as a function of both MVO_2_ (Fig. 4B) and RPP (Supplementary Fig. 1B), which was further increased by scavenging of ROS through administration of MPG + TEMPOL, particularly during exercise, suggesting a net vasodilator influence of ROS in DM + HFD + CKD swine. This resulted in lower cv sO_2_, while cv pO_2_ failed to reach statistical significance (Fig. 4D, F, Supplementary Fig. 1D, F). In contrast, ROS scavenging had no effect on the myocardial oxygen balance in Normal swine either as a function of MVO_2_ (Fig. 4A, C, E) or as a function of RPP (Supplementary Fig. 1A, C, E), suggesting that there is either no role of ROS in the regulation of coronary resistance vessel tone in Normal swine or that vasodilator and vasoconstrictor ROS are well balanced.

### Endothelin receptor blockade during exercise

**Fig. 5:**
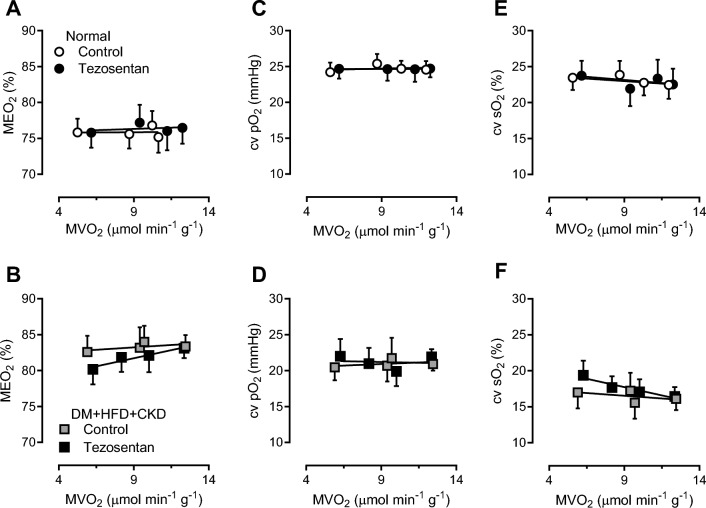
In vivo effects of dual endothelin receptor ET_A_ and ET_B_ blockade (tezosentan) on myocardial oxygen balance at rest and during exercise in Normal and DM + HFD + CKD swine. *MDO*_*2*_ myocardial oxygen delivery, *MVO*_*2*_ myocardial oxygen consumption, *MEO*_*2*_ myocardial oxygen extraction, *cv sO*_*2*_ coronary venous oxygen saturation, *cv pO*_*2*_ coronary venous partial oxygen pressure. Values are mean ± SEM. *P ≤ 0.05 by two-way ANCOVA for repeated measures. Normal *n* = 8, DM + HFD + CKD *n* = 7

Endothelin plasma concentrations were significantly higher in DM + HFD + CKD compared to control (Table 1). However, dual blockade of ET_A_ and ET_B_ receptors with tezosentan did not significantly affect MEO_2_ at rest or during exercise in either group (neither as a function of myocardial oxygen consumption (Fig. 5) nor as a function of the rate–pressure product (Supplementary Fig. 2)), while mean arterial pressure was significantly reduced by tezosentan but to a similar extent in both groups (Supplementary Table 3), consistent with our previous findings [90].

### In vitro microvascular function

**Fig. 6:**
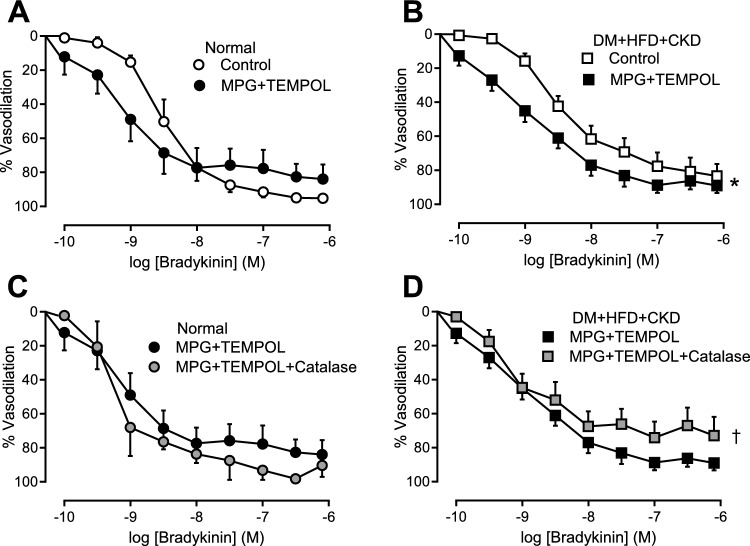
In vitro effects of ROS scavenging on bradykinin-induced vasodilation in isolated coronary small arteries from Normal and DM + HFD + CKD swine. Bradykinin (BK; Normal *n* = 6, DM + HFD + CKD *n* = 13), MPG + TEMPOL (Normal *n* = 6, DM + HFD + CKD *n* = 13), MPG + TEMPOL + catalase (Normal *n* = 5, DM + HFD + CKD *n* = 9). Values are mean ± SEM. *P ≤ 0.05 MPG + TEMPOL vs. BK; †*P* ≤ 0.05 vs. MPG + TEMPOL by two-way ANOVA for repeated measures

To delineate the mechanisms underlying the differences in coronary vasomotor control in response to ROS scavenging observed in vivo, coronary small artery segments were studied in vitro*.* The endothelium-dependent vasodilation to bradykinin was blunted in DM + HFD + CKD segments, while sodium nitroprusside (SNP)-induced relaxation was not different from Normal (Supplementary Fig. 3). Scavenging of ROS (MPG + TEMPOL) improved endothelium-dependent vasodilation to bradykinin in small coronary arteries of DM + HFD + CKD, while it had no significant effect in Normal arteries (Fig. 6A, B). Addition of catalase to MPG + TEMPOL impaired relaxation to bradykinin (as compared to MPG + TEMPOL) in the DM + HFD + CKD group, but not in Normal, consistent with a vasodilator effect through H_2_O_2_ after antioxidant treatment with MPG + TEMPOL (Fig. 6C, D).

### Circulating sphingolipids and ceramides

**Fig. 7:**
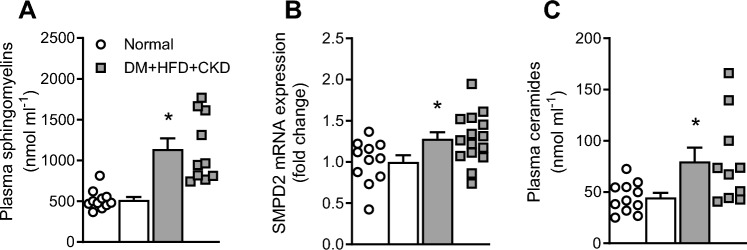
Plasma sphingolipid and ceramide concentrations and left ventricular expression of sphingomyelin phosphodiesterase 2 (SMPD2) in Normal (*n* = 11) and DM + HFD + CKD (*n* = 10) swine. Values are mean ± SEM *P ≤ 0.05 Normal vs. DM + HFD + CKD by unpaired Student’s *t* test

Increased production of H_2_O_2_ by mitochondria as a consequence of increased intracellular ceramide concentrations has been proposed to have a vasodilator effect on coronary vasomotor tone in patients with coronary artery disease [30]. Total plasma concentrations of sphingolipids were significantly higher in DM + HFD + CKD compared to Normal swine (Fig. 7A). The increased levels of ceramides in plasma of DM + HFD + CKD animals could be attributed to an increased hydrolysis of sphingolipids toward ceramides as supported by an increased expression of sphingomyelin phosphodiesterase 2 (SMPD2) in left ventricular tissue of DM + HFD + CKD compared to Normal swine (Fig. 7B, C).

## Discussion

**Fig. 8:**
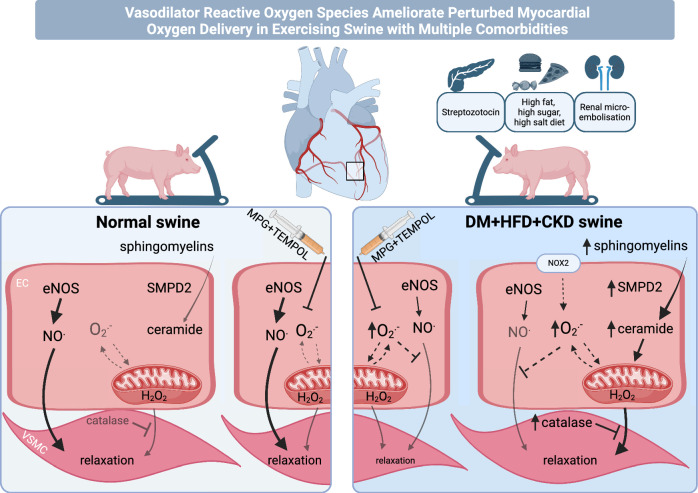
NO contributes to exercise-induced coronary vasorelaxation in healthy swine, which is lost in swine with diabetes, hypercholesterolemia and chronic kidney disease (DM + HFD + CKD). In diseased swine increased NOX2 and ceramide production together with (mitochondrial) ROS augment mitochondrial derived H_2_O_2_. Despite increased catalase activity, H_2_O_2_ mitigates coronary vasoconstriction induced by loss of NO. ROS scavengers MPG + TEMPOL did not change the vascular tone in Normal animals but increased vascular tone in DM + HFD + CKD animals

We previously reported that 5 months of DM, HFD and CKD resulted in impaired myocardial oxygen delivery associated with loss of NO bioavailability and increased oxidative stress in female swine [78, 89]. In the present study, we investigated the effect of ROS scavenging on myocardial oxygen delivery in female swine with DM + HFD + CKD. The main findings (Fig. 8) were that: (1) elevated myocardial 8-iso-PGF2α and circulating TBARS were observed in DM + HFD + CKD compared to Normal swine, indicating increased reactive oxygen species; (2) ROS scavenging with MPG and TEMPOL resulted in an impaired myocardial oxygen delivery at a given level of oxygen consumption, reflecting an increase in coronary resistance vessel tone in DM + HFD + CKD swine in vivo, while ROS scavenging had no effect in Normal swine; (3) catalase, in the presence of ROS scavenging, impaired the vasodilator response to bradykinin in isolated coronary small arteries of DM + HFD + CKD but not Normal swine, suggesting the involvement of H_2_O_2_; (4) plasma sphingolipids and ceramide concentrations were elevated in DM + HFD + CKD swine (5); in vivo dual blockade of endothelin ET_A_ and ET_B_ receptors with tezosentan did not significantly affect myocardial oxygen delivery in either DM + HFD + CKD or Normal swine. The implications of these findings will be discussed below.

The pathophysiology of CMD remains incompletely understood, while its involvement in INOCA and heart failure with preserved ejection fraction in patients with cardiovascular risk factors is increasingly acknowledged [1, 9, 36, 39, 40, 49]. A better understanding of the effect of common risk factors on the coronary microvasculature at both the tissue and cellular level could provide new therapeutic targets to delay or even cease the CMD progression. There are clear sex differences, not only in the prevalence and severity of CMD [1, 15], but also in cardiometabolic risk profile and its effect on the development of CMD [48, 57, 67] as well in the treatment efficacy of ischemic cardiovascular disease [28]. It is important to carefully consider sex as a biological variable in research into CMD, although sex-related differences in the effects of cardiac ischemia were absent in adult Göttingen mini-swine [44]. Nevertheless, given the higher prevalence of CMD in females [1, 15], in the present study, we choose to use female swine only. An inherent limitation to the use of Yorkshire × Landrace swine is their rapid growth, while the strain of swine can also affect vascular responsiveness and cardiovascular (patho-) physiology [25, 45]. We choose to use swine of 3–4 months of age (20–25 kg) at the beginning of the study that were  ~ 8–9 months of age (100–120 kg) toward the end of the study. Swine are sexually mature at an age of 5–6 months and breeding is recommended when sows are  ~ 120 kg [72]. Thus, the swine in the present study can be considered juvenile to adolescent swine. Hence the protective role of estrogen is likely to still be limited in our swine, and they presumably also have a high biological potential for protection and repair [5]. Therefore, extrapolation of our findings to (elderly) male subjects and/or postmenopausal women needs to be done with caution.

We have previously documented CMD in female swine exposed for 5 months to DM + HFD + CKD by a reduction in coronary flow reserve in the absence of significant atherosclerosis in the epicardial coronary arteries, in combination with perturbations in myocardial oxygen delivery at rest and during exercise [78, 89]. The present study further shows that exhaustion of coronary flow reserve during exercise is accompanied by a higher MEO_2_ and lowering of the myocardial net lactate consumption in DM + HFD + CKD, but not Normal swine. Altogether, these data strongly suggest myocardial perfusion abnormalities during exercise, indicative of CMD in DM + HFD + CKD swine. Our previous study showed that CMD in this model was characterized by endothelial dysfunction, with a loss of NO signaling in vivo and impaired endothelium-dependent NO-mediated vasodilation in vitro. As endothelial nitric oxide synthase (eNOS) expression, uncoupling and phosphorylation were not altered and increased oxidative stress was documented, in the present study we investigated the hypothesis that NO bioavailability was reduced due to ROS-mediated scavenging of NO [88, 89].

ROS are involved in cellular homeostasis [81], with different ROS having different, sometimes opposite functions. Thus, superoxide induces vasoconstriction whereas H_2_O_2_ induces vasodilation in the murine [24], porcine [63] and human [93] coronary vasculature. The ROS concentrations and interconversions are tightly regulated via numerous enzymatic and non-enzymatic mechanisms. When this regulation falls short, oxidative stress arises. Oxidative stress has been shown to be involved in the development of cardiovascular disease [7, 23, 66, 81]. Similar to observations in patients with multiple comorbidities, increased levels of left ventricular 8-iso-PGF2α (marker for lipid peroxidation), and reduced left ventricular myocardial total antioxidant capacity were documented in the present study. This is suggestive of an imbalance between oxygen radical production and antioxidant mechanisms. These findings are also consistent with increased myocardial superoxide levels in our previous work in the same animal model [78].

Given the reduced antioxidant capacity, the vasoconstrictor properties of superoxide and the impaired myocardial perfusion in swine with DM + HFD + CKD, our initial hypothesis was that ROS scavenging would improve the perturbed myocardial oxygen balance in this swine model. Surprisingly, ROS scavenging using the combination of the glycine-derived antioxidant MPG and the SOD mimetic TEMPOL, increased myocardial oxygen extraction, indicative of a further impairment of myocardial perfusion and oxygen delivery. These data strongly suggest that ROS have an overall vasodilator effect on the coronary microvasculature in vivo in female swine with DM + HFD + CKD, and hence that the vasodilator effect of H_2_O_2_ outweighs the vasoconstrictor effect of superoxide. These data are consistent with data in murine, canine and porcine hearts, showing that H_2_O_2_ contributes to metabolic vasodilation through activation of K_v_ channels [6, 22, 24].

Our in vivo data are partially corroborated by our studies in isolated coronary small arteries. In apparent contrast with our in vivo data, ROS scavenging with MPG + TEMPOL improved the endothelium-dependent vasodilation to bradykinin in small coronary arteries isolated from DM + HFD + CKD animals, suggesting a net vasoconstrictor effect of ROS. However, further addition of catalase, which converts H_2_O_2_ to water and oxygen, attenuated this vasoconstriction, which is consistent with our in vivo observation that H_2_O_2_ exerts a vasodilator effect on the coronary microvasculature of swine with DM + HFD + CKD. The lack of the effects of the (intercellular) signaling and stimuli from the surrounding tissues and the blood on the coronary tone control of isolated small coronary arteries may have influenced the balance between vasodilator and vasoconstrictor ROS. For example, flow-induced vasodilation was, in contrast to bradykinin-induced vasodilation, shown to increase H_2_O_2_ formation derived from superoxide-generated from mitochondrial respiration in vascular endothelial cells [51, 59], and the contribution of H_2_O_2_ to flow-induced dilation was larger in coronary small arteries from young, as compared to old female rats [41]. Furthermore, the hyperoxic conditions during wire myography may favor generation of superoxide, overwhelm endogenous antioxidant defense mechanisms and thereby result in alterations of the balance between superoxide and H_2_O_2_. In support of this, Wong, et al. showed increased superoxide levels in the hyperoxic buffer. The superoxide scavenger Tiron converted this superoxide to H_2_O_2_, increasing the bradykinin-induced vasodilator response [54, 91]. The increased vasodilation with Tiron was most likely the consequence of both a reduction of superoxide concentration and an increase in H_2_O_2_. Conversely, Batenburg, et al*.* observed that modulation of H_2_O_2_ did not affect the bradykinin-induced vasorelaxation in healthy human isolated coronary small arteries [2]. Similarly, we observed the effect of catalase only in coronary small arteries of swine with DM + HFD + CKD, suggesting that CMD promotes generation of H_2_O_2_. Moreover, our pilot data in isolated healthy porcine coronary arteries showed similar responses to bradykinin either with or without MPG + TEMPOL irrespective of the use of normoxic (MOPS) or hyperoxic (Krebs) buffer (Supplementary Fig. 4). Taken together, even though H_2_O_2_ may have another origin in vivo and in vitro, our small coronary artery wire-myography data are consistent with H_2_O_2_ as a vasodilator in the coronary microvasculature in swine with DM + HFD + CKD.

Possible sources of ROS production in the presence of multiple risk factors include NADPH oxidase (NOX), uncoupled eNOS, xanthine oxidoreductase XOR and dysfunctional mitochondria as will be further discussed below. In the present study, higher expression of NOX2 was measured in the left ventricular myocardial tissue of DM + HFD + CKD animals. This finding is in agreement with recent data reported in coronary microvascular angina patients showing higher serum levels of soluble NOX2-derived protein which correlated with a lower NO bioavailability and increased levels of endothelin-1 (ET-1) [52] as also observed in the present animal model [90]. Loss of NO [18] and increased oxidative stress [84] cause upregulation of ET-1 in the vasculature. This may either directly or through activation of NOX2 and concomitant vascular ROS production impair coronary vasodilation and thereby contribute to the impaired oxygen delivery in the myocardium of animals with comorbidities. However, in the present study, in vivo dual blockade of endothelin receptors ET_A_ and ET_B_ with tezosentan in the DM + HFD + CKD swine model did not alter myocardial oxygen delivery, rendering direct ET-1- mediated vasoconstriction or ET-mediated ROS production unlikely to be the main mechanism involved in the impaired myocardial oxygen delivery in the DM + HFD + CKD swine.

Another possible source of ROS may be uncoupled nitric oxide synthase, which produces superoxide instead of NO, and has been shown to contribute to endothelial dysfunction in diabetic and hypercholesterolemic patients [35, 79] as well as in preclinical hyperglycemic animal models [58]. eNOS abundance, coupling and phosphorylation measured in bulk myocardium were not changed in the DM + HFD + CKD swine compared to Normal [88], suggesting that uncoupled eNOS was not a major ROS source in this model. However, since the measurements were performed in bulk myocardial tissue abundant in cardiomyocytes and fibroblasts, small changes in endothelial eNOS function may be masked. In contrast, eNOS was previously reported to be uncoupled in our DM + HFD + CKD swine model [78]. Although not readily explained, a possible reason for this inconsistency could be a strain difference between our previous and present studies.

Xanthine oxidoreductase (XOR) is another potential source of ROS in the vascular endothelium. Dependent on the form of XOR, the enzymatic reaction is accompanied by NADH production (xanthine dehydrogenase, XDH) or superoxide and hydrogen peroxide generation (xanthine oxidase, XO) [3, 55]. XDH to XO conversion is stimulated by H_2_O_2_ and this can be prevented by NO [56]. Although the gene expression of XOR was unchanged in the myocardium of the DM + HFD + CKD swine, the lower bioavailability of NO in these animals may provide a feed-forward mechanism producing ROS [88]. Moreover, under hypoxic conditions, generation of H_2_O_2_ by XO is augmented over superoxide [10]. XO-derived superoxide has been reported to reduce NO-dependent arteriolar vasodilation in rats receiving high fat diet [27]. In patients, XOR activity was increased in the presence of comorbidities, including type 2 diabetes, renal disease and metabolic syndrome (reviewed in [68]). Thus, it is possible that also in our swine model, XOR activity may have been higher despite unaltered gene expression. Interestingly, downregulated XDH has been reported and associated with capillary rarefaction in a HFD + CKD swine model [26].

In the present, as well as our previous study in the same model, reduced cardiac oxygen utilization efficiency was demonstrated, consistent with mitochondrial uncoupling [89]. In several swine models, high fat diet and chronic kidney disease led to cardiac mitochondrial dysfunction [34, 60], with RNA sequencing results showing that mitochondrial genes related to aerobic respiration and oxidative phosphorylation being downregulated [11]. These data confirm findings from an earlier study, indicating that CKD in particular was responsible for cardiac mitochondrial uncoupling leading to increased mitochondrial H_2_O_2_ production, while high fat diet aggravated myocardial superoxide production and fibrosis [60]. Similarly, increased mitochondrial-derived ROS have been reported in patients with other cardiovascular risk factors, e.g., obesity [20] and diabetes [8, 32]. Furthermore, it should be noted that the net effect of ROS is not only determined by their production, but also by their degradation. Indeed, genes related to reactive oxygen species metabolic processes, including GPX1 and peroxiredoxin (PRDX) 1 and 4, were upregulated in three healthy vs three HFD + CKD swine [11]. Conversely, in the present study, the mitochondrial antioxidant pathways (GPX, SOD2 and SOD3 [61]) were not different in the myocardium of DM + HFD + CKD and Normal swine. Although the unchanged GPX1 is in apparent contrast with the mRNA seq data from Chade and co-workers [11], showing a lower GPX1 expression, they also did not observe differences in mRNA expression of SOD1, 2 or 3 between CKD and control swine.

In the present study, expression and activity of catalase was increased in the myocardial tissue of DM + HFD + CKD swine. H_2_O_2_ has repeatedly been reported to upregulate catalase (reviewed in [46]), and thus increased catalase activity may be a consequence of increased H_2_O_2_ production. Moreover, catalase overexpression was protective against aging-induced cardiomyopathy in mice [73, 85] and diabetic cardiomyopathy in rats [92]. Possibly, either dependent on specific disease etiology or as a consequence of compartmentalization [94], data on catalase activity in diabetes and metabolic syndrome patients are inconsistent, as increased, reduced and unchanged catalase activity in patients compared to controls are reported [46]. Based on this knowledge, we can conclude that increased myocardial catalase activity and expression is a compensatory mechanism, suggesting increased myocardial H_2_O_2_ concentrations.

Our in vitro and in vivo results jointly support the hypothesis that H_2_O_2_ acts as a (ROS-mediated) vasodilator, balancing the lower bioavailability of NO in the animals with comorbidities. Indeed, a switch from NO- mediated vasodilation to H_2_O_2_ has previously been reported in small coronary arteries of patients with coronary artery disease [4], the increased H_2_O_2_ production being associated with increased ceramide concentrations in plasma of these patients [30]. In the dysfunctional endothelium of these patients, increased activation of neutral sphingomyelinases (NSmase1 or SMPD2) can convert the circulating sphingomyelins resulting in increased intracellular ceramide concentrations, subsequently leading to increased mitochondrial H_2_O_2_ production [31]. Increased sphingomyelin concentrations have indeed been associated with multiple cardiovascular diseases (reviewed in [31, 43, 77]). In agreement with these findings, in the present study, higher circulating concentrations of sphingolipids were found in DM + HFD + CKD swine, most likely induced by the high fat diet [12]. Additionally, cardiac SMPD2 expression was increased, potentiating intracellular ceramide generation. Inflammatory factors such as TNFα and ET-1 were shown to increase SMPD2 activity [13, 62, 77], likely being one of the mechanisms involved in the present study. Future studies, directly modulating ceramide with upregulation of ceramidase or inhibition of ceramide synthesis, are required to determine a causal role for ceramides in the increased (mitochondrial) H_2_O_2_ production.

## Future perspectives

Although oxidative stress is associated with CMD [9, 29, 42], up till now, antioxidant therapies did not alleviate CMD. This has been attributed to pharmacokinetic limitations, incompatible drug distribution to the sites of ROS production and the disrupting effect on ROS vital for normal homeostasis and cardiac protection [23, 37, 81]. In light of our findings, future studies should take into account that therapies targeting mechanisms such as ROS might in fact have a detrimental effect by counteracting endogenous compensatory (rescue) mechanisms. Specific inhibitors of sphingomyelinases and ceramide synthases may provide attractive drug targets to prevent mitochondrial uncoupling [71]. However, high selectivity and titration will be necessary, since increased levels of sphingolipids as a result of inhibition of ceramide synthases also have been shown to induce mitochondrial dysfunction [74]. Recently, therapy targeting the adiponectin pathway has been shown to restore NO-mediated vasodilation in small arteries isolated from patients with coronary artery disease, successfully reversing H_2_O_2_ to NO-dependent vasodilatation [14]. Using adiponectin to promote the breakdown of ceramide [38] and production of NO by eNOS [33] might therefore be a more promising therapeutic strategy.

## Conclusion

The present study investigated the effect of ROS scavenging on myocardial oxygen balance at rest and during exercise in premenopausal female swine with common cardiovascular risk factors resulting in coronary microvascular disease. ROS scavenging did not result in improvement in myocardial oxygen delivery in the DM + HFD + CKD swine, but revealed a possible switch from NO to H_2_O_2_-mediated vasomotor control, also supported by in vitro analysis of small coronary arteries. The increased H_2_O_2_ production was likely mediated by increased ceramide production. These results could partly explain the negative results of ROS scavenging therapies in different clinical trials and provide deeper insights into the pathophysiology of coronary microvascular disease in the presence of common risk factors, leading to the identification of possible therapeutic targets for these patients. Furthermore, whether our results could be extrapolated to postmenopausal and/or male subjects remains to be investigated.

## Supplementary Information

Below is the link to the electronic supplementary material.

Supplementary file1 (DOCX 133 KB)

## Data Availability

Data are available from the authors upon reasonable request.
