# Supplementary material for: Vasodilator reactive oxygen species ameliorate perturbed myocardial oxygen delivery in exercising swine with multiple comorbidities
Source: Basic Res Cardiol. 2024 May 25;119(5):869–87. doi: 10.1007/s00395-024-01055-z (PMC11461570; doi:10.1007/s00395-024-01055-z)
Supplement: Supplementary file 1 — Supplementary file1 (DOCX 133 KB) [file 395_2024_1055_MOESM1_ESM.docx]

**Supplemental data**

**Vasodilator Reactive Oxygen Species Ameliorate Perturbed Myocardial Oxygen Delivery in Exercising Swine with Multiple Comorbidities**

*Running title:* ROS Improve Myocardial O_2_ Delivery in Multimorbidity Swine

*R.W.A. van Drie^1,2^ BSc, *J. van de Wouw^1^ MD, PhD, L.M. Zandbergen^1,5^ MSc, J. Dehairs^3^ PhD, J.V. Swinnen^3^ PhD, M.T. Mulder^2^ PhD, M.C. Verhaar^4^ MD, PhD, A. MaassenVanDenBrink^2^ PhD, D.J. Duncker^1^ MD, PhD, ^#^O. Sorop^1^ PhD, and ^#^D. Merkus^1,5,6^ PhD

^1^Division of Experimental Cardiology, Department of Cardiology, Erasmus University Medical Center, Rotterdam, The Netherlands, ^2^Division of Vascular Medicine and Pharmacology, Department of Internal Medicine, Erasmus University Medical Center, Rotterdam, The Netherlands; ^3^Laboratory of Lipid Metabolism and Cancer, Department of Oncology, KU Leuven - University of Leuven, Leuven, Belgium;

^4^Department of Nephrology and Hypertension, University Medical Center Utrecht, Utrecht, The Netherlands; ^5^Walter Brendel Center of Experimental Medicine (WBex), University Clinic Munich, 81377 LMU Munich, Germany, German; ^6^Center for Cardiovascular Research (DZHK), Munich Heart Alliance (MHA), Partner Site Munich, 81377 Munich, Germany

*^#^ Both authors have equal contributions to this paper

Corresponding authors:

Daphne Merkus PhD

Division of Experimental Cardiology

Department of Cardiology, Thoraxcenter

Erasmus University Medical Center,

PO Box 2040, 3000 CA Rotterdam

The Netherlands

Telephone: +31 10 7038066

E-mail: [d.merkus@erasmusmc.nl](mailto:d.merkus@erasmusmc.nl)

**Supplemental Table 1.** Primers used for qPCR

| Gene | Forward primer | Reverse primer |
| --- | --- | --- |
| CAT | TGCCACCGGCAACTATCCCT | TCGCTGTGAGGCCAAACCTTG |
| GPX | ACCGACCCCAAGTTTATCAC | CATCAGGTGTTCCTCCACA |
| HPRT1 | GGACTTGAATCATGTTTGTG | CAGATGTTTCCAAACTCAAC |
| NOX2 | CCGCATTGTTGGCGACTGGA | CCCGTCCACAGCGATCTTAGG |
| NOX4 | ACCAGATGCTGGGGGATTGTG | CCTCGAAGGTAAGCCAGGAGTGT |
| RPL13A | TGGCCAAGCAGGTACTTCTG | GTATTCATGCGCTTGCGGAG |
| SMPD2 | TGGAGAGCTTCGACCTTGTT | TGCTGGGTGAATTCCTGGAT |
| SOD1 | TGGGCAATGTGACTGCTGGC | TCATGGACCACCATTGTGCGG |
| SOD2 | CTTGCAGATTGCCGCTTGTT | CGGCGTATCGCTCAGTTACA |
| SOD3 | CAGACACACTCTCCGCTTCT | AGACCTTCGGGGTAAATGG |
| XOR | AGCAGGGAGGGTGAGTTTTT | GTCTTGAGGGCTGAGATGGT |

CAT, catalase; GPX, glutathione peroxide; HPRT1, hypoxanthine phosphoribosyltransferase 1; NOX2, NADPH oxidase2; NOX4, NADPH oxidase4, RPL13A, ribosomal protein L13; SMPD2, sphingomyelin phosphodiesterase 2; SOD1, superoxide dismutase 2; SOD2, superoxide dismutase 2; SOD3, superoxide dismutase 3; XOR, xanthine oxidoreductase.

**Supplemental Table 2.** *In vitro* evaluation of oxidative regulation in Normal and DM+HFD+CKD swine

|  | n | Normal | | | n | DM+HFD+CKD | | |
| --- | --- | --- | --- | --- | --- | --- | --- | --- |
| SOD1 (fold change) | 11 | 1.00 | ± | 0.04 | 16 | 1.08 | ± | 0.05 |
| SOD2 (fold change) | 11 | 1.00 | ± | 0.13 | 14 | 0.98 | ± | 0.07 |
| SOD3 (fold change) | 8 | 1.00 | ± | 0.14 | 8 | 1.05 | ± | 0.15 |
| XOR (fold change) | 11 | 1.00 | ± | 0.18 | 16 | 0.67 | ± | 0.11 |
| GPX (fold change) | 11 | 1.00 | ± | 0.10 | 11 | 0.98 | ± | 0.10 |

Left ventricular tissue mRNA expression as fold change mean ± SEM relative to Normal, corrected for housekeeping genes hypoxanthine phosphoribosyltransferase 1 (HPRT1A) and ribosomal protein L13a (RPL13A): SOD, superoxide dismutase; XOR, xanthine oxidoreductase; GPX, glutathione peroxidase 1; glutathione. **P* ≤ 0.05 Normal vs DM+HFD+CKD.

**Supplemental Figure 1.** In vivo effects of ROS scavenging in rest and during exercise in Normal and DM+HFD+CKD swine. The relationship between the rate pressure product (RPP, the product of heart rate and systolic arterial pressure) and myocardial oxygen extraction (MEO_2_, **A,B**), partial oxygen pressure (cv pO_2_, **C,D**), coronary venous oxygen saturation (cv sO_2_, **E,F**) in Normal and DM+HFD+CKD swine at rest and during exercise, in control conditions and in the presence of ROS scavenging with MPG+TEMPOL. Values are mean±SEM. *P ≤ 0.05 by two-way ANCOVA for repeated measures. Normal n = 5, DM+HFD+CKD n = 8

|  |  |  |  | **Standing** | | | **Exercise** | | | | | | | | |  | ANOVA *P* value | | |
| --- | --- | --- | --- | --- | --- | --- | --- | --- | --- | --- | --- | --- | --- | --- | --- | --- | --- | --- | --- |
|  |  |  | **n** |  | | | **2 km h^-1^** | | | **3 km h^-1^** | | | **4 km h^-1^** | | |  | Tezosentan | Group | Tezosentan *Group |
| Heart rate | Normal | Control | 9 | 114 | ± | 4 | 165 | ± | 10* | 204 | ± | 13* | 228 | ± | 11* |  |  |  |  |
| (beats min^-1^) |  | Tezosentan | 9 | 134 | ± | 6‡ | 187 | ± | 11* | 213 | ± | 13* | 237 | ± | 10* |  | .038 |  |  |
|  | DM+HFD+CKD | Control | 11 | 121 | ± | 9 | 162 | ± | 12* | 186 | ± | 11* | 203 | ± | 12* |  |  | .312 |  |
|  |  | Tezosentan | 11 | 127 | ± | 6 | 178 | ± | 10* | 199 | ± | 10* | 221 | ± | 10* |  | .120 | .237 | .952 |
|  |  |  |  |  |  |  |  |  |  |  |  |  |  |  |  |  |  |  |  |
| MAP | Normal | Control | 9 | 90 | ± | 4 | 93 | ± | 3 | 97 | ± | 2 | 101 | ± | 3 |  |  |  |  |
| (mmHg) |  | Tezosentan | 9 | 79 | ± | 2‡ | 85 | ± | 2‡ | 90 | ± | 3*‡ | 91 | ± | 4*‡ |  | .000 |  |  |
|  | DM+HFD+CKD | Control | 10 | 88 | ± | 1 | 88 | ± | 4 | 93 | ± | 3 | 94 | ± | 4 |  |  | .130 |  |
|  |  | Tezosentan | 10 | 79 | ± | 4 | 81 | ± | 3 | 86 | ± | 3 | 96 | ± | 3 |  | .002 | .202 | .781 |
|  |  |  |  |  |  |  |  |  |  |  |  |  |  |  |  |  |  |  |  |
| CI | Normal | Control | 8 | 136 | ± | 8 | 196 | ± | 10* | 202 | ± | 12* | 222 | ± | 13* |  |  |  |  |
| (mL min^-1^ kg^-1^) |  | Tezosentan | 8 | 153 | ± | 13 | 197 | ± | 15 | 211 | ± | 19 | 238 | ± | 17* |  | .800 |  |  |
|  | DM+HFD+CKD | Control | 11 | 136 | ± | 17 | 169 | ± | 16 | 186 | ± | 15 | 195 | ± | 13* |  |  | .059 |  |
|  |  | Tezosentan | 11 | 137 | ± | 12 | 187 | ± | 13* | 203 | ± | 14* | 210 | ± | 10* |  | .110 | .182 | .800 |
|  |  |  |  |  |  |  |  |  |  |  |  |  |  |  |  |  |  |  |  |
| CBF | Normal | Control | 9 | 1.17 | ± | 0.08 | 1.68 | ± | 0.14* | 1.93 | ± | 0.15* | 2.19 | ± | 0.16* |  |  |  |  |
| (ml min^-1^ g^-1^) |  | Tezosentan | 9 | 1.37 | ± | 0.07 | 1.87 | ± | 0.13* | 1.99 | ± | 0.12* | 2.12 | ± | 0.15* |  | .234 |  |  |
|  | DM+HFD+CKD | Control | 11 | 1.41 | ± | 0.12 | 1.87 | ± | 0.20* | 1.97 | ± | 0.17* | 2.15 | ± | 0.17* |  |  | .403 |  |
|  |  | Tezosentan | 11 | 1.39 | ± | 0.11 | 1.98 | ± | 0.15* | 2.15 | ± | 0.16* | 2.29 | ± | 0.19* |  | .312 | .485 | .917 |
|  |  |  |  |  |  |  |  |  |  |  |  |  |  |  |  |  |  |  |  |
| Haemoglobin | Normal | Control | 9 | 9.9 | ± | 0.4 | 10.8 | ± | 0.6 | 11.0 | ± | 0.6 | 11.2 | ± | 0.6 |  |  |  |  |
| (g dl^-1^) |  | Tezosentan | 9 | 9.5 | ± | 0.5 | 10.7 | ± | 0.5 | 11.6 | ± | 1.0 | 12.3 | ± | 0.9 |  | .823 |  |  |
|  | DM+HFD+CKD | Control | 11 | 8.7 | ± | 0.2† | 10.1 | ± | 0.3* | 10.2 | ± | 0.4* | 10.9 | ± | 0.3* |  |  | .019 |  |
|  |  | Tezosentan | 11 | 9.5 | ± | 0.6 | 9.2 | ± | 0.5† | 10.1 | ± | 0.5 | 10.8 | ± | 0.7 |  | .325 | .024 | .676 |
|  |  |  |  |  |  |  |  |  |  |  |  |  |  |  |  |  |  |  |  |
| Arterial sO_2_ | Normal | Control | 9 | 98 | ± | 1 | 98 | ± | 1 | 98 | ± | 1 | 97 | ± | 1 |  |  |  |  |
| (%) |  | Tezosentan | 9 | 98 | ± | 1 | 97 | ± | 1 | 98 | ± | 1 | 96 | ± | 1 |  | .362 |  |  |
|  | DM+HFD+CKD | Control | 11 | 99 | ± | 1 | 97 | ± | 1 | 98 | ± | 1 | 97 | ± | 1 |  |  | .912 |  |
|  |  | Tezosentan | 11 | 98 | ± | 1 | 97 | ± | 1 | 97 | ± | 1 | 98 | ± | 1 |  | .733 | .670 | .697 |
|  |  |  |  |  |  |  |  |  |  |  |  |  |  |  |  |  |  |  |  |
| MVO_2_ | Normal | Control | 8 | 5.2 | ± | 0.5 | 8.7 | ± | 1.1 | 10.2 | ± | 1.1* | 10.6 | ± | 1.4* |  |  |  |  |
| (µmol min^-1^ |  | Tezosentan | 8 | 6.2 | ± | 0.6 | 9.4 | ± | 1.0 | 11.2 | ± | 1.6 | 12.2 | ± | 2.0 |  | 0.923 |  |  |
| g^-1^) | DM+HFD+CKD | Control | 7 | 5.9 | ± | 0.8 | 9.4 | ± | 2.2 | 9.7 | ± | 1.4 | 12.4 | ± | 0.7* |  |  | .966 |  |
|  |  | Tezosentan | 7 | 6.3 | ± | 0.8 | 8.2 | ± | 1.2 | 10.0 | ± | 1.5* | 12.4 | ± | 2.0* |  | 0.584 | .801 | 0.840 |
|  |  |  |  |  |  |  |  |  |  |  |  |  |  |  |  |  |  |  |  |
| RPP | Normal | Control | 9 | 132 | ± | 7 | 201 | ± | 13* | 268 | ± | 22* | 317 | ± | 18* |  |  |  |  |
| (10^-2^ [mmHg |  | Tezosentan | 9 | 146 | ± | 10 | 225 | ± | 19* | 270 | ± | 21* | 304 | ± | 14* |  | .009 |  |  |
| beats min^-1^]) | DM+HFD+CKD | Control | 10 | 139 | ± | 15 | 199 | ± | 21 | 238 | ± | 22* | 259 | ± | 21* |  |  | .405 |  |
|  |  | Tezosentan | 10 | 135 | ± | 8 | 206 | ± | 13* | 242 | ± | 15* | 274 | ± | 16* |  | .635 | .135 | .716 |

**Supplemental Table 3.** Hemodynamics at rest and during exercise under control conditions and in the presence of endothelin receptor blockade with tezosentan in Normal and DM+HFD+CKD swine.

MAP, mean arterial pressure; CI, cardiac output per kg of bodyweight; CBF, coronary blood flow per gram of myocardium; sO_2_, oxygen saturation; MVO_2_, myocardial oxygen consumption; RPP, rate pressure product. Values are mean ± SEM. **P* ≤ 0.05 versus corresponding standing; †*P* ≤ 0.05 versus corresponding Normal; ‡*P* ≤ 0.05 versus corresponding Control by three-way ANOVA for repeated measures and post-hoc analysis with least significant difference correction.

**Supplemental Figure 2.** In vivo effects of dual endothelin receptor ET_A_ and ET_B_ blockade (tezosentan) in rest and during exercise in Normal and DM+HFD+CKD swine. The relationship between the rate pressure product (RPP, the product of heart rate and systolic arterial pressure) and myocardial oxygen extraction (MEO_2_, **A,B**), partial oxygen pressure (cv pO_2_, **C,D**), coronary venous oxygen saturation (cv sO_2_, **E,F**) in Normal and DM+HFD+CKD swine at rest and during exercise, in control conditions and in the presence of ROS scavenging with MPG+TEMPOL. Values are mean±SEM. *P ≤ 0.05 by two-way ANCOVA for repeated measures. Normal n = 8, DM+HFD+CKD n = 7

**
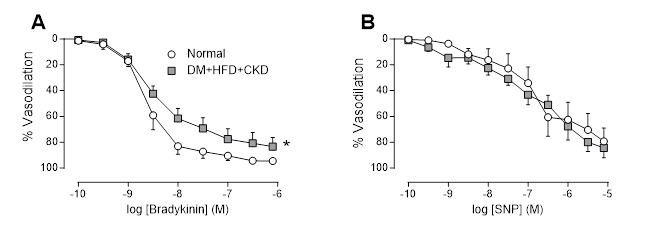
**

**Supplemental Figure 3.** *In vitro* effects of endothelial dependent vasodilator bradykinin (Normal n = 5, DM+HFD+CKD n = 13) and endothelium independent vasodilator sodium nitroprusside (SNP; Normal n = 5, DM+HFD+CKD n = 11). Values are mean ± SEM **P* ≤ 0.05 Normal vs DM+HFD+CKD by two-way ANOVA for repeated measures.

**Supplemental Figure 4.** Pilot data comparing *in vitro* bradykinin-induced vasodilatation with and without ROS scavenging (MPG+TEMPOL) in four healthy coronary arteries using Krebs or MOPS-buffer (composed of the following (in mmol/l): 145.0 NaCl, 4.7 KCl, 1.17 MgSO_4_ · 7H_2_O, 2.0 CaCl · 2H_2_O, 1.2 Na H_2_PO_4_ · H_2_O, 5.0 glucose, and 2.0 pyruvate; pH 7.35 ± 0.02). Values are mean ± SEM.
